# The Riddle of the Good Faith Purchaser

**DOI:** 10.1093/ojls/gqae037

**Published:** 2024-11-14

**Authors:** Michael J R Crawford

**Keywords:** law and economics, property law, commercial law

## Abstract

A purchaser unwittingly buys stolen goods. The owner from whom they were stolen demands their return. The purchaser refuses. How should the law resolve their dispute? This article argues that the law’s primary objective in resolving disputes between owners and good faith purchasers should not be to achieve ‘justice’ between the parties but to disincentivise theft. With some categories of goods, it is difficult to see how the legal attribution of liability can achieve this end. However, where goods are amenable to registration, the rules of good faith purchase can discourage theft by conditioning an owner’s success over a good faith purchaser on the fact of prior registration. In the absence of a register, there seems little to choose between the parties. However, because the favoured party will frequently be a monopolist, the danger of holdouts warrants employing innovations from auction theory, the effect of which is to force the parties to reveal otherwise private information about their subjective valuations of the disputed goods.

## 1. Introduction

An owner (*O*) identifies an asset stolen from her by a thief (*T*) in the possession of a good faith purchaser (*P*). *P* refuses *O*’s request to return the asset, so *O* sues *P* in conversion. Who should win? The most obvious answer seems to be *O*, for the simple reason that she is the owner. However, because ‘owner’ is simply the designation we attached to the person with the best legal claim, this argument assumes the very thing it seeks to prove. A better argument is that *O* should prevail because it is intrinsic to the concept of ownership that it is impervious to divestment by non-consensual transfer.^1^ Whilst it is true that no system of property could permit *T* to acquire good title from *O*, it does not follow that *O* must therefore prevail over *P*. Such a proposition is belied by the notorious diversity of solutions to this universal problem across jurisdictions both ancient and modern.^2^ Even English law, which recognises the general principle that ‘property rights survive dispositions by non-owners, even where the recipient is an innocent purchaser for value’,^3^ admits numerous exceptions to it.^4^

What makes such disputes particularly difficult is the absence of the ‘correlatively structured’ reasons for liability that are central to the corrective justice paradigm of private law adjudication.^5^ Whilst *O* and *T*, and *P* and *T*, are bilaterally linked as the ‘doer’ and sufferer’ of the same injustice, the same cannot be said of *O* and *P*.^6^ To adapt Weinrib’s nomenclature, the ‘normative positions’ occupied by *O* and *P* are not ‘mirror images of each other’.^7^ To the contrary, the only thing linking *O* and *P* is their shared status as victims of *T*’s wrongdoing. Because *T* is generally either unidentifiable or otherwise ‘not worth the powder and shot’,^8^ one sympathises with McFarlane’s view that the need to distribute the loss between *O* and *P* raises an ‘impossibly difficult question’^9^ for private law.

However, the problem takes on a different complexion if we abandon the corrective justice paradigm and reconsider the concept of loss. Whilst either *O* or *P* will have been made *worse off* by *T*’s actions, we can only say there has been a *loss* if we ignore the corresponding gain to *T*. As discussed in section 2, the involuntary transaction effected by *T* appears to be a transfer, the effects of which are purely distributive. If this is true, then what is *T*’s great sin? The answer is that his actions are never costless. From the value obtained by *T*, we must subtract not only the loss to *O*, but also the sum of the costs to *T* of committing the theft, to *O* of guarding against *T*’s depredations, to *P* of determining whether his vendor has good title and to society of the deadweight losses caused by distorted investment decisions. Whilst theft may be profitable to *T*, it is always socially wasteful.

Because the true loss caused by *T*’s conduct is the social loss occasioned by the existence of theft as an enterprise, the question that the law should answer is not which allocation of liability between *O* and *P* accords with some notion of corrective or distributive justice, but which is most likely to suppress theft by minimising the returns to *T* from selling stolen goods. The argument advanced in section 3 is that there is no obvious way of doing this. Indeed, when the asset is of low value or difficult to identify, it is unlikely that the legal attribution of liability between *O* and *P* will have any behavioural effect at all.

However, when the stolen asset is valuable *and* readily identifiable, *O*’s likely investment in search means that the law’s solution to her dispute with *P* will alter the parties’ behaviour. Crucially, such assets are also amenable to registration. Building on the work of Bell and Parchomovsky,^10^ section 4 argues that registers are the most effective non-punitive tool for reducing the returns of theft. Where a register exists, the law should maximise its ‘obstructive effect’ by conditioning *O*’s success on prior registration of the disputed goods.

What, then, should the law do in the absence of a register? As a matter of overall welfare, the law should be indifferent about the outcome. Because the dispute between *O* and *P* is nominally zero-sum, the only effect of the legal rules is to shift, rather than reduce, the misfortune. In the absence of reliable information about the highest-valuing user, there is no compelling reason to favour *O* over *P*, or *vice versa*. Either outcome is justifiable on the ground that each fulfils the law’s basic function of definitively resolving private disputes. However, because the favoured party will often be in the position of a monopolist and thus tempted to hold out, section 5 argues that there is reason to depart from private law’s traditional binary resolution of such disputes by employing strategies from auction theory that force the parties to reveal their otherwise private valuations.

## 2. The (Economic) Problem, Identified

### A. Transfers Versus Losses

Why does the law care about the involuntary transfer from *O* to *P* effected by *T*? From a rights-based perspective, the answer is the absence of *O*’s consent. From the perspective of efficiency, however, the answer is less clear.^11^ Although many economic analyses of the good faith purchaser problem condition liability on the cheapest cost-avoider principle,^12^ it is not clear that there is any loss to avoid. Whilst an accident victim’s pain and suffering is a deadweight loss that avails no one,^13^*O*’s loss is accompanied by a corresponding gain to *T*, making the involuntary transaction a pure transfer, the only effect of which is to redistribute wealth from *O* to *T*.^14^ Indeed, if *P* is the higher-valuing user, *T*’s actions will have increased overall welfare. Why, then, does the law regard the dispute between *O* and *P* as a problem to be solved? Why not simply ignore *O*’s claim and accord *de jure* status to the *de facto* allocation of resources brought about by *T*’s actions?

### B. The Problem of Rent Seeking

One reason is that *P* may not be the higher-valuing user, in which case the involuntary transaction will have created a real loss equal to the difference between *O*’s and *P*’s subjective valuations. Is this a good reason for the law to intervene and reverse the transaction by ordering *P* to deliver up the asset or pay damages? The answer to this question is probably ‘no’. Even if it could be demonstrated that *O* were the higher-valuing user, judicial intervention will only be justified if the difference in subjective valuation exceeds the total cost of reversing the transfer. A rule favouring *O*s over *P*s would only be welfare-increasing if the former value the asset more than the latter *and* the average of that difference exceeds the average cost of reversing the transfer. Because *P*s are not volunteers, the average difference in valuation between *Os* and *Ps* is likely to be small, meaning that this requirement will seldom be satisfied.

In fact, even where the cost of reversing the transaction is less than the difference in subjective valuation, an attempt by *O* to reverse the transfer is probably still wasteful. This is because the true cost of procuring transfers of wealth is the positive opportunity cost of the resources employed in doing so.^15^ For instance, if both *O* and *P* value the goods at £100, then it is privately worthwhile but socially wasteful for *O* to expend £20 to procure a transfer from *P*. The picture looks different if *O* values the good at £121. In this case, spending £20 to reverse the involuntary transfer yields a total gain of £1. However, if that £20 would have yielded a £30 gain if invested in some positive-sum enterprise, *O*’s behaviour is still socially wasteful even though an order that *P* deliver up the goods to *O* is not a pure transfer.

## 3. Can the Rules of Good Faith Purchase Minimise the Social Costs of Theft?

If the law of good faith purchase should aim to discourage *O* from investing scarce resources with alternative uses in a zero-sum game, then the obvious solution should be to favour *P* in a dispute with *O*. If this is correct, then the puzzle is why so many systems favour some version of the *nemo dat* rule that dictates precisely the opposite outcome. Whilst this might be attributed to ignorance on the part of the system’s architects or to a willingness to tolerate inefficiency in service of some other goal,^16^ another explanation is that permitting rent seeking by *O* will be efficient if it discourages the greater evil of larceny by *T*. To understand why, it is necessary to discuss the social costs of theft.

### A. The Social Cost of Theft

If the involuntary transaction between *O* and *P*, ‘brokered’ by *T*, is a pure transfer, then one may wonder why a prohibition on non-consensual transfers is a human universal. The answer, unsurprisingly, is that theft is not a mere transfer. The involuntary transaction from *O* to *P* could only be a transfer if *P*’s valuation were at least as high as *O's and T*’s acts, including both the theft from *O* and the sale to *P*, were costless. Whilst the first condition may be satisfied, the second never is. Just as *O* must expend resources with alternative uses to procure a re-transfer from *P*, so too must *T* expend resources to procure a notionally zero-sum transfer from *O*. Whilst *T*’s actions may be privately profitable, they are socially wasteful.

The social loss caused by *T* is not limited to the opportunity cost of the resources invested in the initial theft. To prevent future depredations by *T*, *O* will invest in countermeasures, prompting *T* to invest in efforts to overcome those countermeasures, and so on. This cycle will continue until the entirety of *T*’s rent is dissipated in this fruitless struggle with *O*.^17^ What begins as an apparently zero-sum transfer to *T* thus ends in a massively wasteful negative-sum arms race.^18^ To these costs must be added the deadweight losses caused by distorted investment decisions.^19^ Instead of devoting her resources to producing the most valuable assets, *O* may instead decide to produce assets that are difficult to steal. Indeed, the simplest way to avoid theft is to produce nothing at all.^20^

Theft could only be efficient if *T* were the higher-valuing user and the costs of his rent seeking were less than the transaction costs of a consensual sale. Given that the express purpose of markets is to reduce transaction costs,^21^ this is unlikely to be true. Even if it were, this requirement is never satisfied in the three-party transactions under consideration because *T*’s costs of stealing from *O* are in addition to, and not in substitution of, the cost of negotiating the consensual sale to *P*.^22^ Indeed, the truth is worse than our three-party schematic suggests. Because *T* specialises in theft rather than resale, there is likely to be one or more ‘fences’ interposed between *T* and *P*. Whilst their existence is testament to the benefits of specialisation in both the licit and illicit economies, it also results in a manyfold increase in the total transaction costs between *O* as the involuntary ‘seller’ and *P* as the final buyer.

Theft not only increases transaction costs by multiplying the number of transacting parties. In a system in which *O* is favoured over *P*, it also increases transaction costs for *P* because the mere existence of theft as ‘a potential activity’ means that *P* must bear both the usual costs of bargaining and the additional verification cost of ensuring that the vendor has good title to pass.

That theft is always a negative sum activity does not detract from the truth of the argument advanced in section 2. After the stolen goods have been sold to *P*, any attempt by *O* to reverse the transaction is rent-seeking behaviour. However, the costs of *O*’s *ex post* rent seeking will be justified if they deter theft *ex ante*. The question is which, if any, solution to the dispute between *O* and *P* will reduce the returns from theft. To answer this question, it is necessary to give a brief account of the economics of crime.

### B. The Simple Economics of Crime

‘Crime pays’ if *T*’s marginal revenue exceeds his marginal costs. Where *T* steals goods for the purpose of resale, his revenue is the resale value of the goods. His costs are the sum of the transaction costs of selling to *P*, the expected value of the sanction^23^ and the opportunity cost of the resources used in the commission of the crime. Maximising the expected value of crime is thus a complex optimisation exercise. Fabricating a false provenance for stolen goods is expensive, but may result in a substantially higher resale price. Devoting more time to planning a crime is costly, but will reduce *T*’s chances of being caught and thus the expected value of the sanction.

How to make theft a losing proposition is also complex. The marginal cost of crime differs from person to person, not merely because some criminals are cleverer than others and thus less likely to be caught, but also because the opportunity costs of crime are not uniform. To people whose skills enable them to command high incomes in the licit economy, most crime has a high opportunity cost. To those without such skills, it is relatively lower.^24^ Moreover, the efficacy of a sanction depends not only on its expected value, but on the relative weighting of the probability of being convicted and the severity of the sanction. Although a 10% chance of a £1000 fine and a 100% chance of a £100 fine have the same expected value, they do not have the same utility.^25^ The deterrent effect of the former is low for someone who is risk tolerant and high for someone who is risk averse, and *vice versa*. To this may be added competing considerations of efficiency and justice. Whilst increasing the severity of the sanction without increasing the probability of apprehension is the cheapest way of increasing its expected cost, severing the link between the gravity of the offence and the sanction undermines its moral legitimacy.^26^

### C. Using the Rules of Good Faith Purchase to Deter Theft

These difficulties aside, crime will be made less attractive by measures that increase *T*’s marginal cost or reduce his marginal benefit, or both.^27^ In principle, the law’s solution to the good faith purchaser problem could assist in the achievement of these goals by allocating liability between *O* and *P* in a way that incentivises *O* to take efficient precautions against theft and *P* to take efficient precautions against buying from thieves. Such precautions would both increase the cost to *T* of stealing from *O* and reduce the price at which he can sell to *P*. Efficiency requires that *O* invest in theft prevention measures until the marginal reduction in the probability of theft multiplied by the asset’s subjective value equals the marginal cost of prevention, and that *P* invest in title verification measures until the marginal increase in the probability of receiving a good title multiplied by the asset’s subjective value equals the marginal cost of verification.^28^

The problem is that this ‘joint-care’ solution to the problem of theft is extremely difficult to achieve.^29^ This is because, as Schwartz and Scott explain,^30^ no title-based solution to the problem of good faith purchase can simultaneously incentivise both *O* and *P* to take optimal precautions. *O* will only take optimal precautions if he cannot recover from *P*, and *P* will only inquire optimally into her vendor’s title if she must relinquish the goods to *O*. Because no categorical title-based system can simultaneously achieve these outcomes, the law finds itself on the horns of a ‘double moral hazard’.^31^ In systems that recognise exceptions to *nemo dat*, this problem is ameliorated by conditioning *P*’s success on proof that her investment in title verification is sufficient to satisfy the good faith standard. However, due to the cost of implementing a more exacting standard, good faith generally only requires that *P* demonstrate ‘honesty in fact’,^32^ which is a weak approximation of the optimality standard and thus insufficient to eliminate her moral hazard.

Because favouring *O* over *P* or *P* over *O* simply substitutes one set of misaligned incentives for another, the only way of ensuring that each party invests optimally is to require that neither wins. However, because the law must select a winner, the relevant question is whether *O*’s optimal investment in precautions against theft or *P*’s optimal investment in title verification is likely to do most to deter theft. There is a general, though tentative, belief that exceptions to *nemo dat* increase returns to theft.^33^ There are several reasons to believe that this is true. First, because *P* need only satisfy the undemanding good faith requirement, *T* need only invest minimally in fabricating a false provenance for the goods. Secondly, a rule that favours *P* largely destroys *O*’s incentive to search, leaving the task of detection entirely in the hands of police who, in the absence of pressure from *O*, may be less than zealous in making enquiries. Indeed, if *O* regards the task of recovering the goods as hopeless, she may only report the theft if it is a requirement of her insurance, should she have it.^34^ The concomitant reduction in search makes it less likely that *T* will be identified and apprehended, thus reducing the expected value of the sanction. Thirdly, a rule that favours *P* may reduce her verification costs, thus reducing the effective price of stolen goods and so increasing demand for them.

Importantly, the magnitude of this final effect is limited by the fact that *P* must still demonstrate her *bona fides*. Thus, as Weinberg has observed,^35^ an alteration in the rules to favour *P* will have no effect on those who knowingly purchase stolen goods and thus could never establish their innocence. Irrespective of the legal regime, such *P*s will manage their title risk by discounting the purchase price. Nor will it affect those who, as a matter of principle, refuse to purchase from vendors who seem suspicious, even if the sale would pass the good faith test. A change from favouring *O* to favouring *P* will only benefit those who suspect the goods of being stolen but can nevertheless satisfy the good faith requirement.^36^ Despite the relatively narrow class of affected sales, we may nevertheless expect a rule that favours *P* to result in *some* increase in demand for stolen goods.

### D. The Limits of (Private) Law

At the cost of some rent seeking by *O*, a rule that favours *O* over *P* should discourage theft by *T*. Because the social cost of *T*’s behaviour is likely to dwarf that of *O’s*, systems concerned with efficiency should resolve disputes between *O* and *P* in favour of the former. However, if this is true, why do so many jurisdictions permit departures from the *nemo dat* rule?

As noted above, possible explanations for favouring *P* over *O* include economic ignorance or the promotion of values other than efficiency. A different, and more plausible, explanation is that the difficulty and cost of locating stolen goods means that the law’s solution to the dispute is unlikely to have any effect on either *O*’s level of rent seeking or *T*’s investment in crime.^37^

*O* will search for stolen goods until the marginal search cost equals the marginal increase in the probability of recovering the goods multiplied by their subjective value to her. In practice, this means that, unless the goods are both extremely valuable *and* readily identifiable, *O* will invest almost nothing in search because she knows that her efforts will be in vain.^38^ On the one hand, this has the salutary effect of both deterring *O* from engaging in wasteful attempts to recover the assets from *P* and, absent insurance, ensuring that she invests optimally in precautions against theft. On the other hand, it also guarantees that *P*’s private level of enquiry will be substantially below the socially optimal level.^39^ In a world in which *O* will not bother to search for stolen goods, *P* will only invest optimally in title verification if, as a matter of principle, he refuses to purchase stolen goods. Whilst such people exist, one cannot smuggle such exogenous ‘moralisms’ into an account that is predicated on rational maximisers. Instead, we must assume that *P* will behave in a manner that is individually rational but ‘socially perverse’.^40^

Because *P*’s underinvestment in verification is reducible to a stubborn fact about the difficulty of finding stolen goods and not the insoluble double moral hazard caused by title rules, it cannot be solved by Schwartz and Scott’s suggestion of replacing title rules with a fault-based negligence standard.^41^ As they explain it:

a ‘tort type’ solution would permit [*O*] to recover involuntarily transferred goods unless she was negligent in protecting them. If she was negligent, then the buyer … can keep the goods. An owner who anticipates being unable to recover the goods if she is negligent will take optimal precautions. And since a negligence rule permits nonnegligent owners to recover stolen goods, the rule retains the owner’s incentive to search optimally for them. This proposed negligence rule would make buyers strictly liable because they would always lose to nonnegligent owners. Repealing rules that protect buyers who purchase in good faith would also raise the inquiry level that buyers would choose.^42^

In a world in which *O* believes that she will never locate the stolen goods, a negligence rule is not required to induce her to take optimal precautions against theft.^43^ Likewise, in a world in which *P* correctly surmises that *O* will not bother to search for stolen goods, favouring *O* over *P*, with or without a negligence requirement, will not alter *P*’s behaviour.^44^

The only way to reconcile the private and socially optimal level of *P*’s enquiry is to favour *O and* charge *P* some additional premium, known as a ‘kicker’, the expected value of which is equal to the difference between the private and socially optimal levels of enquiry.^45^ Though it may be effective, the kicker’s punitive quality makes it extremely controversial. Due to the unlikelihood of *O* ever finding *P*, reconciling the private and socially optimal level of *P*’s level of enquiry would require a kicker that is many multiples of the value of goods purchased in good faith.

If the efficient solution to the problem of theft requires the use of sanctions, then the most obvious solution is to allow the criminal law to more effectively punish *T*. Whether this is done by raising the severity of the sanction, the probability of its imposition or both, this is beyond the purview of private law.

## 4. Of Registration

The foregoing discussion can be reduced to the following. The crucial loss caused by *T*’s forced transaction is not the misfortune suffered by the disfavoured party. Nor is it the difference in subjective valuations between *O* and *P*. Rather, it is the social cost of theft as an enterprise. If the law’s solution to the problem of good faith purchase should aim to achieve anything, it is deterring theft by *T*. However, the prohibitive cost of locating and proving ownership of the stolen goods makes it unlikely that the allocation of liability between *O* and *P* will achieve this end. Given this, *O* and *P* will always behave *as if* the law favours *P*, irrespective of the position *de jure*. The question considered below is whether this is invariably true, or whether the law’s resolution of disputes between *O* and *P* will elicit salutary behavioural changes in respect of some categories of goods.

### A. Unique and Valuable Goods

Recall that *O* will search for stolen goods until the marginal search cost equals the marginal increase in the probability of recovering the goods multiplied by their subjective value to her. She will thus invest minimally in searching for both low value goods *and* valuable goods that are difficult to identify. A valuable good may be difficult to identify because it is ubiquitous and homogeneous or because it is rare and distinctive but can be made anonymous by modification or reduction to its component parts. For instance, distinctive gold jewellery can be melted down, diamonds recut and cars ‘chopped’. Even though the whole is generally worth more than the sum of its parts, the diminution in value caused by dismembering the asset is often more than offset by the reduction in the likelihood of detection.

In some cases, however, this strategy is not viable because altering the stolen asset renders it worthless. A mangled Renoir is a worthless piece of canvas;^46^ a chopped Ferrari Daytona is a pile of rusting steel. If the asset is distinctive, and it is in *T*’s interest to maintain its distinctive qualities, investment in search by *O* may yield a high marginal increase in the probability of recovery. Where *P* knows that *O* is likely to search for the stolen goods, the *de jure* rules for resolving any dispute between them will influence their investment in title verification and precautions against theft, respectively. Crucially, valuable and distinctive chattels not only encourage search, they are also amenable to registration. The argument advanced below is that, outside of the criminal law, registration is the most effective means of deterring *T* from stealing goods for the purpose of resale. Where a register is viable, the law ought to resolve disputes between *O* and *P* in the way that is most likely to encourage *O* to register and *P* to consult the register prior to purchase.

### B. The Virtues of Registration

For the vast majority of human history, the only means of broadcasting ownership information was through some conspicuous and socially accepted act of possession.^47^ The benefit of the possession ‘heuristic’^48^ is that acts of possession are cheap, simple to perform and, particularly in small and homogeneous societies, seldom misunderstood.^49^ In Merrill’s words, possession allows us to ‘successfully navigate through daily life without significant conflict over the thousands of items of value that cross our paths’.^50^ Despite these benefits, it becomes misleading when ownership and possession diverge.^51^

As Epstein notes,^52^ the most obvious way of solving the problem of ‘ostensible ownership’^53^ is to require that an owner also possess. Though it seems implausible, this has been the common law’s traditional method for dealing with divided interests in personal property. The seminal authority is *Twyne’s Case*,^54^ in which Twyne accepted a conveyance of Pierce’s sheep as security for a debt, but allowed Pierce to retain possession so that he could shear them. Another of Pierce’s creditors was subsequently permitted to seize the sheep in execution on the ground that Twyne’s failure to take possession made the conveyance from Pierce fraudulent and thus void against his other creditors. Since *Twyne’s Case*, it has been ‘considered an almost conclusive badge of fraud for the secured creditor to leave his debtor in possession of the security’.^55^

As is true of all exceptions to *nemo dat*, the rule in *Twyne’s Case* substantially reduces the verification costs of non-owners. However, it comes at a high price. Conditioning the *in rem* quality of a right on the fact of possession forecloses on the possibility of estates, incorporeal hereditaments, bailments, secured lending and agency arrangements in which rights lurk unseen behind the possessor.^56^ Although outweighed by the benefits, the cost of allowing possession to diverge from title is the surplus from the trades thwarted by the cost of determining whether rights in an asset are held by someone other than the possessor.

The virtue of a system of registration is that it allows a right holder to cheaply broadcast information about her rights, whether she is in possession or not. The concomitant reduction in a potential purchaser’s title verification costs facilitates otherwise losing trades. For present purposes, the great benefit of registration is that it removes the information asymmetry between *T* and *P*. The asymmetry arises because *T* has a great deal of information about the provenance of the article offered for sale whilst *P* has very little. Because *T* will, *ex hypothesi*, answer any questions about his title dishonestly, the cost to *P* of accurately assessing the quality of *T*’s title is generally prohibitive. However, where ownership information is available on a register, the marginal cost to *P* of verifying *T*’s title is trivial.

Whilst commentators generally stress a register’s ‘facilitative’^57^ function of promoting transactions otherwise blocked by title verification costs, an underappreciated virtue of registers is what Bell and Parchomovsky call their ‘obstructive function’.^58^ If the register reveals that *T* is not the owner and *P* is a ‘person of principle’, then no sale will occur. Even if *P* is not influenced by such moralisms,^59^ she will still insist on a steep discount on the purchase price to reflect the infirmity of *T*’s title.^60^ In either case, the presence of cheaply accessible title information will reduce *T*’s revenue, thus making theft a less desirable activity compared to its alternatives in the licit economy. Moreover, because *P* will be aware that *T* has no right to sell the goods, she may also report *T* to the police, thus increasing the chances of apprehension and thus the expected value of the sanction.

### C. The Limits of Registration

Registration is the most effective private law mechanism for minimising the returns on *T*’s investment in theft. Moreover, where *O*’s rights appear on the register, the resolution of disputes between *O* and *P* is trivial. *O* must prevail for the simple reason that *P* will have constructive knowledge of *O*’s rights, thus negating his good faith status. In the presence of a register, there can be no disputes between owners and *good faith* purchasers.

Registration is not, however, a panacea. First and foremost, this is because registration is only appropriate for a relatively narrow class of assets. Because the marginal cost of searching the register is positive and does not vary with the value of the asset to be registered, registration is inappropriate for low value items. No one who purchases a bottle of milk from the corner shop would bother to consult the register of milk owners.^61^ Even if the expected marginal benefit of consulting the register exceeds its marginal cost, a register will only be efficient if the sum of the gains from the register’s facilitative and obstructive functions exceeds the cost of its creation and maintenance, including the cost of training lawyers and laymen in its use. This is unlikely to be satisfied for low value assets, or assets that are perishable or consumable and thus unlikely to be traded more than once.^62^

Registration will also be inappropriate for an asset whose appearance can be modified by *T* so that it no longer resembles its description in the register.^63^ To use Bell and Parchomovsky’s terminology, a viable system of registration requires a ‘stable alignment between registered information and legal rights’.^64^ Irrespective of its value, an asset will be unsuited to registration if it can be melted down, recut, reduced to its components parts or have its unique identifying marks excised. Thus, with some exceptions, registration will be inappropriate for many items of jewellery, precious stones, cars and electrical equipment.

Moreover, whilst registers discourage simple larceny, they encourage identity theft.^65^ As Bell and Parchomovsky note,^66^ thieves whose *modus operandi* is to steal the identity of the registered owner succeed *because* of the register’s basic facilitating function. Whilst a register will always encourage some substitution between crimes, the magnitude of this effect will depend on the register’s design features.^67^ It will be less pronounced under a system of pure ‘recordation’ and more pronounced under a system of registration that creates a state-guaranteed title.^68^ Because the latter system gives legal effect to an otherwise void instrument, *P* will underinvest in title verification, making it easier and thus more profitable for *T* to perpetrate identity fraud.^69^

A final, and underappreciated, objection to registers is their tendency to redistribute income by dispersing their costs across all taxpayers but confining their benefits to those who use them. This redistribution is likely to be regressive because, for the reasons discussed above, assets that are amenable to registration tend to be owned by the wealthy. Take a register for ownership interests in artwork. Because a painting is valuable, durable and valueless if disfigured, it is a prime candidate for registration. By eliminating questions about title, a register can benefit both vendors and purchasers. Imagine that the reserve prices of a vendor and a prospective purchaser are £90 and £100, respectively. If the purchaser must incur £9 to verify the quality of the vendor’s title, she will offer no more than £91 for the painting. Because verification costs are a deadweight loss and not a transfer, they do not benefit the vendor. However, if a register reduces those costs to virtually zero, then the purchaser may offer £100 for the painting, effectively increasing the value of the vendor’s asset by £9. Alternatively, if the vendor accepts £91, the register will have saved the purchaser £9. That the register has made the vendor and purchaser better off is, however, cold comfort for those who do not buy and sell art, but must subsidise the diversions of the wealthy dilettantes who do.

### D. Registers as Private Goods

What, then, should the law do in the face of these objections? One answer is that the state should charge registration and search fees to ensure that the costs of creating and operating the register are internalised by its users and not borne by taxpayers generally.^70^ A second, and simpler, answer is ‘nothing’. This is because a state-funded register is wasteful for low value assets and unnecessary for valuable assets because it is a service that the market can provide to those who value it.

Whilst most systems of registration with which we are familiar are supplied by the state, registers are not inherently ‘public goods’. A public good is one that, due to its non-rival and non-excludable nature, cannot be supplied to one person without being supplied to everyone. Classic examples are lighthouses and police forces.^71^ Public goods are thus a problem of market failure caused by ‘positive’ externalities. Whilst people would be willing to pay their share of the price required to provide the good, the absence of an effective exclusionary mechanism creates a powerful incentive to freeride, the result of which is that no one pays and the good is not provided. Although information is the non-rival good *par excellence*, registers containing information about the ownership of personal property are not inherently public goods because information is an ‘excludable’ resource. A physical register can be placed in a desk under lock and key; an electronic register can be placed behind a password-protected paywall.

Proof that this is not merely an economist’s fantasy is the existence of the ‘Art Loss Register’, maintained by International Art and Antique Loss Register Ltd,^72^ which is the world’s largest database of lost and stolen works of art, rare books and antiquities.^73^ The company also hosts a separate database for lost, stolen and counterfeit watches.^74^ According to its own figures, its artwork database includes more than 700,000 items, and is searched more than 450,000 times annually.^75^ The Art Loss Register is a for-profit service that charges both the victims of theft to register their items in the database and prospective purchasers to search it. At present, the company charges a registration fee of £15 and a search fee of £70.^76^ Whilst a £70 fee might deter casual browsing, it is a negligible sum for purchasers who are considering buying an expensive item with a questionable provenance. International Art and Antique Loss Register Ltd is not the only company to offer such a service. In addition to providing recovery services, Art Recovery International LLC is currently creating a private database of stolen, looted or otherwise missing artwork.^77^ Given the ease with which such global, online databases can be both compiled and searched, we should expect their coverage to rapidly grow.^78^

### E. Using the Rules of Good Faith Purchase to Create Comprehensive Private Registers

Because the costs of private registers are internalised by those who choose to use them, they do not regressively redistribute wealth and are thus politically neutral. However, their voluntary nature also makes them less comprehensive than their state-sanctioned counterparts, limiting the efficacy of both their facilitative and obstructive functions. Indeed, an incomplete register of stolen goods may actively facilitate the sale of stolen goods because *T* can point to the absence of an entry on the register as evidence of his authority to sell.

One might think that the benefits of a register’s obstructive function would induce owners of valuable art to voluntarily register. This, however, is not necessarily true. As others have noted,^79^ the creation of a voluntary and comprehensive register of *title* to artwork is thwarted by a pervasive belief that registration will attract the unwanted attention of revenue authorities.^80^ Even a comprehensive register of *lost or stolen* items is difficult to create due to a tension between the interests of *O* before and after the theft. *Ex ante*, *O* benefits from registration because the obstructive effect of a comprehensive register reduces the returns to theft and thus minimises the chances that one will occur. *Ex post*, however, registration reduces *O*’s chances of recovery because any act that publicises the theft will deter *T* from offering the asset for resale in the secondary markets that *O* is able to monitor.^81^

Lest it be dismissed as purely theoretical, this strategy of secrecy was successfully employed by the Guggenheim Museum in *Solomon R Guggenheim Foundation v Lubell*.^82^*Lubell* concerned a Chagall gouache that was stolen from the Museum by a mailroom employee and ultimately sold to Mrs Lubell, a good faith purchaser. The Museum candidly admitted that its decision to conceal the theft from both law enforcement and other cultural institutions was driven by a concern that any form of publicity would drive the stolen work underground. Its strategy was vindicated when the work, which Mrs Lubell had exhibited both publicly and privately, came to the attention of a Sotheby’s agent who was a former Museum employee. The Court of Appeals of New York decided in favour of the Museum, rejecting Lubell’s argument that the statute of limitations imposes a duty of ‘reasonable diligence’ on the party against whom time runs. The Court instead affirmed the hitherto accepted view that time did not begin to run until Lubell had refused the Museum’s demand for the return of the work.^83^ Amongst other reasons, the Court was concerned that the creation of a more purchaser-friendly rule would make New York City a centre for the trade in stolen artwork.^84^

This divergence between *O*’s interest *ex ante* and *ex post*, so vividly illustrated by the facts in *Lubell*, is an example of a collective action problem essentially identical to that caused by the payment of ransoms to kidnappers.^85^ Whilst it is in the hostage’s interest to buy his freedom, paying a ransom is socially undesirable because it makes kidnapping lucrative and thus encourages more of it.^86^ However, if our primary concern is reducing the social costs of theft, then the law should incentivise the actions that make theft less likely, rather than those that make recovery more likely.^87^

In the presence of a register, creating the socially optimal incentives requires a limited departure from *nemo dat*. This suggestion has also been made by Bibas,^88^ who argues that if *O* promptly notifies police and registers her loss on a computerised register or database, she should always prevail in any subsequent dispute against *P*. On the other hand, if *O* fails to take these steps, *P* should prevail. Whilst there is little to choose between the parties if *O* does not register and *P* does not search, favouring *P* has the salutary effect of encouraging the creation of a comprehensive register by incentivising *O* to register her lost or stolen work on pain of forfeiting title to *P*.^89^

This solution is easily accommodated into the existing law as a defence to *P*’s liability in conversion to *O*. To prevail in a dispute against *O*, *P* must discharge the evidentiary burden of proving both the existence of a register and the failure by *O* to register the disputed item. Whilst such a defence is bound to cause disputes about the requisite notoriety of the register in some cases, this is a small price to pay for the obstructive effect of registration. Importantly, because such a rule will encourage the creation of ever larger and more comprehensive registers, disputes about the notoriety of the register will rapidly diminish.

Admittedly, if the register only records ownership information about lost or stolen assets, even perfect coverage will not prevent all fraudulent sales. It would not, for instance, prevent unauthorised sales by rogue agents or other bailees who have possession with *O*’s consent. Capturing these cases would require a true register of title which, for the reasons noted above, is unlikely to be created voluntarily.^90^ Nevertheless, one should never let the perfect be the enemy of the good, and a comprehensive, private register of lost or stolen works would represent a dramatic improvement over the *status quo*.

## 5. Divide and Conquer: Strategies for Revealing Private Information

### A. The Indifference Hypothesis

This, of course, provides no assistance in resolving disputes between *O* and *P* in the absence of a register. If neither outcome creates obviously undesirable incentives *ex ante*, then whom should the law favour?

What is striking about comparative studies of good faith purchase is the diversity of solutions to a problem that afflicts every system that permits the alienation of private property rights. In a recent study, Chang identified at least 23 distinct approaches across 247 jurisdictions.^91^ The stubborn resistance to convergence apparent in Chang’s survey of modern law is consistent with the historical pattern of variety identified by Levmore in jurisdictions dating from the Code of Hammurabi to modern English, American and European law.^92^

An obvious conclusion to draw from this diversity of solutions is that none is appreciably better than any other. The answer to the question posed above is thus: ‘either party’. Weinberg, for instance, sensibly observes that the longevity of the *nemo dat* rule in common law jurisdictions is at least *prima facie* evidence of its efficiency.^93^ By parity of reasoning, however, the persistence of rules that favour *P* must also be *prima facie* evidence of the tolerable efficiency of the opposite rule.^94^ As Rogers has noted:

Civil law systems tend to provide greater protection to purchasers of stolen goods than do common law systems, but it would require an impressive bit of blind faith in instrumentalist creed to suppose that the rules on stolen goods have caused a significant difference in the economic development of say, England and France.^95^

If nothing else, we can be confident that no powerful group or coalition has ‘rigged’ the system by entrenching a rule that is inefficient but favours the interests of its members. This is because favouring *O* over *P* or *P* over *O* is distributively neutral.^96^ What people gain whilst wearing their ‘owner’ hat they lose whilst wearing their ‘purchaser’ hat.^97^ Because, *ex ante*, no one knows which role she will occupy when the axe falls, no one is tempted to favour a rule that is socially expensive but privately favourable.^98^ As Epstein observes, *O* and *P* ‘are behind the veil of ignorance, not by philosophical stipulation, but as a simple matter of fact’.^99^ Indeed, these mixed motives are not confined to the fact that *P*s become *O*s when title passes, and thus change from favouring a system that recognises exceptions to *nemo dat* to one that rigidly enforces it. Because the price that *P* is prepared to pay *O* for some asset depends in part on the security of his ownership once title has passed, and because the value of ownership to *O* depends in part on her ability to someday sell it, it is not even straightforwardly true that *O*s prefer unyielding enforcement of the *nemo dat* rule whereas *P*s prefer extensive exceptions to it.^100^

Levmore has posited a ‘functional approach’ to the issue that

suggests that variety in the rules of different legal systems may be found either where different rules lead to similar behavioral effects or where reasonable lawmakers could disagree over which of these rules is best … I argue that variety in the treatment of the good-faith purchaser of stolen property can be linked to the second cause of variety, that is, the difficulty of discerning the best solution to a hard question.^101^

This conclusion is unconvincing when applied to low value goods. In such cases, the better explanation for variety is that, for the reasons discussed in section 3D, the attribution of liability between *O* and *P* is unlikely to elicit any behavioural changes at all. A more difficult question is whether the ‘indifference hypothesis’ still holds for goods that are valuable and readily identifiable.

As argued in section 3, a rule that favours *O* over *P* is probably more theft-retardant than the alternative. Nevertheless, there are two reasons to believe that the magnitude of this effect is likely to be small. First, at least for valuable and unique goods, the *nemo dat* rule incentivises *P* to invest in title verification by incentivising *O* to search for the stolen goods. However, this is only true in the absence of insurance. Once insurance in introduced, the contractual allocation of risk between *O* and his insurer replaces the default legal allocation as the dominant source of behavioural influence. If *O* is indemnified against theft, he will have little incentive to search. Due to the pervasive problem of moral hazard, he may also underinvest in precautions against theft.^102^ Whilst an insurer may be subrogated to *O*’s claims against *P* and *T*, there is no guarantee that it will invest optimally in search either. Depending on the competitiveness of the market, it may instead succumb to its own moral hazard by simply writing the asset off and spreading the loss amongst the members of the insurance pool.

Secondly, if all societies prohibit the form of non-consensual acquisition we call ‘theft’, then we should also expect them to prefer rules that, *ceteris paribus*, tend to deter it. That societies have not converged on *nemo dat* as the preferred solution to disputes between *O* and *P* strongly suggests that its ability to deter theft is overstated.

Despite these observations, the danger of strategic behaviour under monopoly conditions provides reason to question the indifference hypothesis in the absence of a register.

### B. Transaction Costs and Holdouts

It was noted in section 2 that a rule which resolved a dispute between *O* and *P* in favour of the lowest valuing-user would create a welfare loss equal to the difference between the parties’ subjective valuations. This, however, will only be true if transaction costs block any voluntary rearrangement of the new baseline created by the legal resolution of their dispute.^103^ Where transaction costs are positive but not prohibitive, the loss caused by an inefficient default rule will not be the difference in subjective valuation between *O* and *P*, but the transaction costs necessary to rearrange it.

These costs appear to be the flaw in the indifference hypothesis. If the law could devise a rule that selected the highest-valuing user with a probability better than chance, then it would obviate the need for the parties to incur the cost of bargaining around the inefficient default in many cases. Although a seemingly obvious point, it is not true. Until science discovers a means of measuring hedons and dolors, bargaining is indispensable. Where efficiency is determined by willingness to pay and not subjective desire, the outcome of the bargaining process is precisely what determines the identity of the highest-valuing user.^104^ To say that the default rule selected the highest-valuing user *ex ante* is simply to say that it correctly predicted the outcome of the *ex post* bargaining process, and not that it avoided the need for it. However, whilst a rule that accurately selected the higher-valuing user would not obviate the need for bargaining, it would ameliorate the problem of misallocation caused by holdouts.

The problem of holdouts is particularly acute in contests between *O* and *P* because, for the reasons discussed in section 3, the disputed goods are likely to be valuable and unique. In the absence of a market for substitutes, the favoured party will find himself in the position of a monopolist and be tempted to hold out for the lion’s share of the surplus created by any trade.^105^ As a matter of efficiency, it does not matter if the favoured party ultimately captures 99 per cent of any surplus created by the exchange. However, the danger is that the favoured party’s behaviour will prevent an exchange, thus transforming the initial allocation of rights into the final allocation.^106^ Whilst an accurate default rule will not obviate the need for the parties to incur generic bargaining costs, it will minimise misallocation caused by failed attempts by the favoured party to ‘extort’ the non-favoured party in the absence of a market for substitutes.

The obvious problem is that of selecting a rule that does better than chance.^107^ Whilst *O* may be the higher-valuing user if *P* were a mere volunteer and *P* would almost certainly be the higher-valuing user if *T* were a mercantile agent,^108^ these examples offer no assistance where *P* has given value and *T* is a thief or fence. One might favour *O* on the ground that her ownership of the goods proves that she placed an above-market valuation on them. After all, if her valuation were below the market price, she would have sold them prior to the theft. Quite apart from the fact that *P* may not have paid the market price, this conclusion only follows in a frictionless universe.^109^ In our world of costly transactions, an equally plausible explanation for her decision to retain the goods is that a mutually beneficial sale at the market price was blocked by transaction costs. Alternatively, one might argue that the ‘endowment effect’ will usually mean that *O* is the higher-valuing user.^110^ Whilst this argument appears promising, it fares no better. Whether the endowment effect creates a difference in average valuations between *O*s and *P*s depends on how quickly one becomes ‘endowed’. Because, as Kahneman, Knetsch and Thaler famously demonstrated,^111^ the effect can be instantaneous, it will very likely affect *O* and *P* equally. Even if the effect were delayed, that the sale to *P* usually precedes the litigation by months, if not years, makes it irrelevant in most disputes.

### C. Strategies for Revealing Private Information

If the holdout problem is to be solved, then it is necessary to find some means of forcing the parties to reveal their private valuations. Unfortunately, this cannot be achieved by simply downgrading the favoured party’s level of protection from a ‘property rule’, subject to injunctive relief, to a ‘liability rule’, subject to an award of damages.^112^

As Calabresi and Melamed observed,^113^ the virtue of a liability rule is that the favoured party cannot hold out under monopoly conditions. A liability rule operates as a call option exercisable at the price set by the court assessing the plaintiff’s damages.^114^ A decision by the option holder to ‘exercise’ it by paying damages rather than returning the goods is proof that his subjective valuation exceeds the quantum of damages. However, it is not also proof that the favoured party values the asset at, or less than, the quantum of damages that she will receive as compensation for its loss. Thus, the danger of under-compensation inherent in a liability rule threatens an inefficient taking by the non-favoured party.^115^ This remains true even when the option price equals the value that *P* paid to *T* for the stolen asset. A good example of this is §281 of the Code of Hammurabi,^116^ which permitted *O* to retake a stolen slave purchased by *P* so long as *O* reimbursed *P* for the price paid by *P*. Because we do not know whether the price which *P* paid for the asset was also her maximum price, we cannot know whether a call option exercised at that price is efficient. If we are to avoid this problem, we need a solution other than favouring *O* over *P* or *vice versa*.

The most obvious way of solving this problem is for the court to auction the asset and divide the proceeds of sale equally between *O* and *P*.^117^ This solution solves the holdout problem by the simple expedient of denying either party the status of ‘preferred claimant’. As Parchomovsky, Siegelman and Thel note, equal division is not only the solution that would be chosen *ex ante* by rational, risk-averse people,^118^ it is also the most morally defensible resolution to a dispute between two innocent parties.^119^

If equal division is such an elegant and obvious solution to the problem, then one wonders why it appears to be confined to Mongolian tribal law.^120^ According to Levmore, the answer is cost.^121^ On his view, the relevant cost is that of judicially valuing the disputed asset. Because, for the reasons discussed above, the market for the disputed asset is likely to be thin, attempts at valuation will be both expensive and speculative.^122^ The proposed solution of an auction avoids the need to value the asset, but does so at the cost of conducting an auction under judicial supervision. If this cost is prohibitive, then what is required is a cheap means of eliciting truthful valuations from the parties. Fortunately, there are several mechanisms for doing this.^123^

The simplest and cheapest is for the court to hold a second price, or ‘Vickrey’, auction.^124^ Under a Vickrey auction, the court would invite sealed bids from *O* and *P* and award the disputed asset to the higher bidder, but at the price bid by the lower bidder. Whilst it appears counterintuitive, telling the truth is both parties’ ‘dominant strategy’^125^ because understating one’s true valuation increases one’s chances of missing out on the prize whilst overstating it risks an unprofitable, and thus pyrrhic, victory.^126^ This is not true of sealed bid auctions in which the winner pays the value of her own bid. Under these conditions, a rational bidder will always bid *less* than her true valuation, since to bid her true valuation will ensure zero surplus should she win.

An alternative to the Vickrey auction is some version of the ‘Texas Shootout’, a model most often used to dissolve partnerships and closely held companies.^127^ Under this arrangement, P1 is asked to nominate a value for his share and P2 is granted both a call option, compelling P1 to sell his share to P2, and a put option, compelling P1 to purchase P2’s share, each of which is exercisable at the valuation nominated by P1 for his own share. By making P1 uncertain as to whether he will be buyer or seller, the Texas Shootout encourages honest self-assessed valuations. Whilst P1 will never be tempted to understate his valuation, the risk of being compelled to purchase P2’s share at his own nominated price negates P1’s usual tendency to overstate it. By combining call with put options, the Texas Shootout avoids both the problem of holdouts caused by property rules *and* potential under-compensation caused by liability rules.

As with the Vickrey auction, the Texas Shootout is easily adapted to the problem under consideration. In a dispute between *O* and *P*, a court can declare that each party holds a 50 per cent undivided interest as tenant in common and then nominate one of the parties as the ‘valuer’, who must declare the value he attaches to the 50 per cent share, and the other as the option holder. The option holder may then either purchase the valuer’s share or force him to purchase her share at the price nominated by him. A decision by the option holder to exercise the call option can only be explained on the basis that she values the undivided share more than the valuer. A decision to exercise the put option can only be explained on the ground that she values the share less than the valuer. Irrespective of which option is exercised, the entirety of the asset will be united in the hands of the highest-valuing user.

Both the Vickrey auction and the Texas Shootout elicit truthful information by placing the parties behind a veil of ignorance. Because, *ex ante*, neither knows whether she will become a buyer or seller *ex post*, neither can know whether nominating a high or low valuation will be to her advantage.^128^ Consequently, each party does best by telling the truth. Although a cheap means of forcing the parties to reveal private information, each is subject to the distributive objection that it allocates 100 per cent of the difference in valuation to one of two *prima facie* innocent parties. In the Vickrey auction, this is because the winner pays the loser’s reservation price, and not her own. In the Texas Shootout, it is because the option holder need not reveal his private valuation, meaning that the surplus cannot be divided because it is known only to himself.^129^

Given the central concern of this article, the obvious response to this criticism is to dismiss it as a question of just distribution about which an efficiency-minded commentator is agnostic. There are, however, two additional responses. The first is that, as noted by Parchomovsky, Siegelman and Thel of ‘take-or-pay’ systems generally,^130^ the person nominated as valuer in the Texas Shootout is guaranteed to receive, at worst, 50 per cent of her subjective valuation of the disputed asset. This is greatly superior to the ‘nothing’ that she *may* receive under a categorical title system, and is also the outcome that she would choose ‘behind the veil’ if, like most people, she is risk averse. The second is that unequal distribution of the surplus is the rule rather than the exception. This is because a Vickrey auction is conceptually identical to the familiar ascending, or ‘English’, auction, in which, as Vickrey noted,^131^ the winning bidder does not pay his own reservation price, but some nominal increment above that bid by the second highest bidder.^132^

## 6. Conclusion

When viewed from an economic perspective, the real riddle of the good faith purchaser is why it is considered a riddle at all. That the transaction is involuntary does not mean that it results in a loss of overall welfare, and even if it could be demonstrated that *O* were the higher-valuing user, the costs of reversing the transfer would overwhelm any welfare gain. If this is correct, then the obvious solution to a dispute between *O* and *P* should be to favour *P*. However, this ignores the social cost of *T*’s behaviour. Even if *P* were the higher-valuing user, the costs of theft would make the forced transaction wasteful overall. If the law is concerned about social welfare, then private law’s primary objective in resolving disputes between *O* and *P* should not be to maximise welfare or ensure justice *inter partes*, but to deter acts of theft by *T*.

The obstacle to this sensible goal is not merely the double moral hazard created by categorical title rules, but the fact that, due to prohibitively high search costs, the legal attribution of liability between *O* and *P* is unlikely to influence the parties’ behaviour. However, this ceases to be true if the asset is valuable and unique. When substantial investment in search by *O* becomes cost-justified and a dispute between *O* and *P* becomes a realistic prospect, the law’s solution to the dispute begins to bite. The question is, what attribution of liability between *O* and *P* would do most to deter *T*? The argument made above was that, because such goods are amenable to registration, the law should maximise a register’s ‘obstructive effect’ by incentivising *O* to register, at the very least, the fact of the theft. This requires the law to favour *O* over *P* unless the former has failed to register.

This leaves a residual category of disputes in which there is no register. In such cases, favouring *O* over *P*, or *vice versa*, seems to be perfectly adequate, but for the problems caused by the temptation of the favoured party to hold out. If the cost of a judicial sale is prohibitive, the law should avoid the potential for misallocation by employing some version of a Vickrey auction or Texas Shootout to elicit honest valuations from the parties.

## Footnotes

1 Honoré regarded this as one of the standard incidents of ownership, describing it as the ‘right to security’. AM Honoré, ‘Ownership’ in AG Guest (ed), *Oxford Essays in Jurisprudence* (OUP 1961) 107, 119–20.

2 See text to n 91.

3 William Swadling, ‘Restitution and Bona Fide Purchase’ in William Swadling (ed), *The Limits of Restitutionary Claims: A Comparative Analysis* (The United Kingdom National Committee of Comparative Law 1997) 79, 80. This is captured by the maxim *nemo dat quod non habet*, generally abridged to ‘the *nemo dat* principle’. The rule is enshrined in the Sale of Goods Act 1979, s 21(1). See generally Michael Bridge, *Personal Property Law* (4th edn, OUP 2015) 197; Law Reform Committee, *Twelfth Report (Transfer of Title to Chattels)* (Cmnd 2598, 1966) para [3].

4 For a comprehensive discussion of the exceptions to *nemo dat*, see Bridge (n 2) 198–224. The most significant exception to *nemo dat*, sales in ‘market overt’, was abolished by the Sale of Goods Amendment Act 1994.

5 Ernest Weinrib, *The Idea of Private Law* (rev edn, OUP 2012) 120–2. For Kant’s rights-based justification of the market overt rule, see Ernest Weinrib, ‘Private Law and Public Right’ (2011) 61 UTLJ 191, 199–203.

6 See also Michael JR Crawford, ‘Contract as Property: Triangles and Tragic Choices’ (2023) 82 CLJ 83, 99; Aruna Nair and Irit Samet, ‘What Can “Equity’s Darling” Tell Us about Equity?’ in Dennis Klimchuk, Irit Samet and Henry Smith (eds), *Philosophical Foundations of the Law of Equity* (OUP 2020) 264, 268.

7 Ernest Weinrib, *Corrective Justice* (OUP 2012) 10.

8
*Moorgate Mercantile v Twitchings* [1977] AC 890, 907 (Lord Salmon). If *T* were identifiable and solvent and could thus be sued by *O* in conversion or by *P* in deceit or for breach of the implied warranty of title, this problem would cause the law little anxiety. See also Richard Epstein, ‘Inducement of Breach of Contract as a Problem of Ostensible Ownership’ (1987) 16 JLS 1, 8.

9 Ben McFarlane, *The Structure of Property Law* (Hart Publishing 2008) 189.

10 Abraham Bell and Gideon Parchomovsky, ‘Of Property and Information’ (2016) 116 Colum L Rev 237.

11 Throughout this article, ‘efficiency’ will refer to the Kaldor–Hicks variant, which simply requires that total gains exceed total losses. Transfers effected by theft can never satisfy the more demanding ‘Pareto’ criterion, which requires that any change to the *status quo* make at least one person better off and no one else worse off. Due to the stringency of the Pareto criterion, economists generally treat Kaldor–Hicks as the default criterion. See Richard Posner, *Economic Analysis of Law* (9th edn, Wolters Kluwer Law & Business 2014) 14–15.

12 That is, if it is cheaper for *O* to prevent the theft than it is for *P* to verify title, then *P* should prevail. On the other hand, if it is cheaper for *P* to verify title than it is for *O* to avoid the theft, then *O* should prevail. See eg Robert Cooter Jr and Thomas Ulen, *Law and Economics* (6th international edn, Pearson 2014) 140–1; Harold Weinberg, ‘Sales Law, Economics, and the Negotiability of Goods’ (1980) 9 JLS 569, 583; Epstein, ‘Inducement’ (n 8) 13–14; Menachem Mautner, ‘The Eternal Triangles of the Law: Toward a Theory of Priorities in Conflicts Involving Remote Parties’ (1991) 90 Mich L Rev 95, 100; Anthony Ogus, *Costs and Cautionary Tales: Economic Insights for the Law* (Bloomsbury 2006) 45–6. There is, however, no consensus on which party is the cheapest cost-avoider. See Barak Medina, ‘Augmenting the Value of Ownership by Protecting It Only Partially: The Market-Overt Rule Revisited’ (2003) 19 J L Econ & Org 343, 346; Cooter and Ulen (idem) 141.

13 Whilst the wrongdoer *may* save the costs of taking reasonable precautions, she does not benefit from her victim’s pain and suffering.

14 See Steven Shavell, ‘Individual Precautions to Prevent Theft: Private Versus Socially Optimal Behavior’ (1991) 11 International Review of Law and Economics 123, 125 (emphasis omitted). On the distinction between losses and transfers, see Cento Veljanovski, *Economic Principles of Law* (CUP 2007) 28–9.

15 Robert Tollison. ‘The Economic Theory of Rent Seeking’ (2012) 152 Public Choice 73, 73. See further Gordon Tullock, ‘Rent Seeking as a Negative-Sum Game’ in James M Buchanan, Robert D Tollison and Gordon Tullock (eds), *Towards a Theory of the Rent Seeking Society* (Texas A&M University Press 1980) 16.

16 Dari-Mattiacci and Guerriero, for instance, argue that the legal response to this question depends upon the jurisdiction’s conception of morality. See Giuseppe Dari-Mattiacci and Carmine Guerriero, ‘Law and Culture: A Theory of Comparative Variation in Bona Fide Purchase Rules’ (2015) 35 OJLS 543, 546.

17 The marginal thief will thus earn nothing from theft. Most models of rent-seeking behaviour assume complete rent dissipation. See eg Richard Posner, ‘The Social Costs of Monopoly and Regulation’ (1975) 83 Journal of Political Economy 807, 809.

18 Gordon Tullock, ‘The Welfare Costs of Tariffs, Monopolies, and Theft’ (1967) 5 Economic Inquiry 224, 231. See also Fred McChesney, ‘Boxed in: Economists and Benefits from Crime’ (1993) 13 International Review of Law and Economics 225, 227–8.

19 McChesney (n 18) 228.

20 In Bentham’s words, ‘I am unwilling to give myself cares which will only be profitable to my enemies’. Jeremy Bentham, ‘Principles of the Civil Code’ in R Hildreth (tr), *Theory of Legislation* (Trubner & Co 1876) 116.

21 Richard Posner, ‘An Economic Theory of the Criminal Law’ (1985) 85 Colum L Rev 1193, 1195–6.

22 See Richard Hasen and Richard McAdams, ‘The Surprisingly Complex Case against Theft’ (1997) 17 International Review of Law and Economics 367, 372–3.

23 Which equals the value of the sanction multiplied by the probability of receiving it.

24 This fact does much to explain the demography of crime. That (almost) everyone can earn something in the licit economy, and that the effort of committing a crime must be traded off against leisure, also explains why deterrence does not require that the expected value of the punishment equal or exceed *T*’s expected benefit. See Gary Becker, ‘Crime and Punishment: An Economic Approach’ (1968) 76 Journal of Political Economy 169, 176–7.

25 ibid 178.

26 Jules Coleman, *Markets, Morals and the Law* (CUP 1988) 154.

27 Economic theories of crime are concerned with proximate rather than ultimate causes. They do not seek to unearth the fundamental reasons for criminality, but instead point out that, as crime becomes more expensive, people commit less of it. See generally James Q Wilson, *Thinking About Crime* (Hachette UK 2013) ch 7.

28 Alan Schwartz and Robert Scott, ‘Rethinking the Laws of Good Faith Purchase’ (2011) 111 Colum L Rev 1332, 1339; Yun-Chien Chang, *Property Law: Comparative, Empirical and Economic Analyses* (CUP 2023) 270.

29 On ‘joint-care’, see Stephen Gilles, ‘Negligence, Strict Liability, and the Cheapest Cost-Avoider’ (1992) 78 Va L Rev 1291, 1308–9; Steven Shavell, *Economic Analysis of Accident Law* (Harvard UP 2009) 9–12, 17–18.

30 Schwartz and Scott (n 28) 1339.

31 ibid, 1339 fn 29.

32 See Sale of Goods Act, s 61(3), which provides that ‘A thing is deemed to be done in good faith within the meaning of this Act when it is in fact done honestly, whether it is done negligently or not’. An identical definition is found in Bills of Exchange Act 1882, s 90. For discussion, see Michael Bridge and others, *The Law of Personal Property* (3rd edn, Sweet & Maxwell 2022) 920–1 [31-025].

33 See eg Steven Shavell, *Foundations of Economic Analysis of Law* (Harvard UP 2009) 52–4; Medina (n 12) 358; Weinberg (n 12) 580. This was the view held by those who applauded the abolition of the market overt exception. See eg Brian Davenport and Anthony Ross, ‘Market Overt’ in Norman Palmer and Ewan McKendrick (eds), *Interests in Goods* (2nd edn, LLP 1998) 337, 352. Interestingly, the Law Reform Committee’s recommendation that the rule be abolished was based on a concern about its uneven application, and not that it facilitated the sale of stolen goods. A majority of the Committee thus recommended replacing market overt with a general good faith purchaser defence applicable to all sales conducted at trade premises or auction. See Law Reform Committee (n 3) para [33].

34 Incentivising *O* to pursue *T* probably explains why jurisdictions that retain the market overt rule create an exception to the exception where *T* is successfully prosecuted. See Sale of Goods Act 1895 (SA), s 24; Sale of Goods Act 1895 (WA), s 24; Sale of Goods Act 1896 (Tas), s 29. On the development of this rule, see Peter M Smith, ‘Valediction to Market Overt’ (1997) 41 Am J Legal Hist 225, 245–7.

35 Weinberg (n 12) 576–7.

36 In Weinberg’s words, *P* must have a ‘colorable claim of innocence resulting from the circumstance of [his] purchases’. ibid 577. By ‘colorable’, Weinberg means ‘plausible’ and not ‘fraudulent’.

37 See also ibid 583–4. Another explanation is that people can only be guided by the law if they are aware of it, and a great many people are (rationally) ignorant of the law. See James Rogers, ‘Negotiability, Property, and Identity’ (1990) 12 Cardozo L Rev 471, 480–2.

38 Recent estimates put the recovery rate for stolen art at somewhere between 3% and 10%. See Robert Ekelund Jr, John Jackson and Robert Tollison, *The Economics of American Art: Issues, Artists and Market Institutions* (OUP 2017) 185.

39 This point seems to be generally acknowledged amongst lawyer-economists. See Schwartz and Scott (n 28) 1361; Shavell, ‘Foundations’ (n 33) 53–4; Weinberg (n 12) 581–2; Medina (n 12) 355.

40 Shavell, ‘Foundations’ (n 33) 53.

41 Schwartz and Scott (n 28) 1338–42.

42 ibid 1340.

43 That is, *O*’s private level of investment in theft prevention is likely to approximate the social optimum irrespective of the legal regime. See also Chang, *Property Law* (n 28) 273–4.

44 The other flaw in the negligence approach is the cost of establishing the appropriate standard of care. See Epstein, ‘Inducement’ (n 8) 14. The Law Reform Committee cited complexity as the basis for rejecting Lord Devlin’s suggestion that the loss caused by *T* be apportioned between *O* and *P* according to relative fault. Law Reform Committee (n 3) paras [9]–[12]. At common law, *O* is not duty-bound to take reasonable care to safeguard her property. See *Moorgate* (n 8) 902–3 (Lord Wilberforce), 925 (Lord Fraser); *Farquharson Brothers v King* [1902] AC 325, 335–6 (Lord Macnaghten); *Central Newbury Car Auctions Ltd v Unity Finance Ltd* [1957] 1 QB 371 380 (Denning LJ). Schwarz and Scott concede that no jurisdiction has adopted their proposal. Schwartz and Scott (n 28) 1371.

45 See Coleman (n 26) 1157–8; Guido Calabresi and A Douglas Melamed, ‘Property Rules, Liability Rules, and Inalienability: One View of the Cathedral’ (1972) 85 Harv L Rev 1089, 1126 fn 70. Presumably, the kicker should be paid to the state rather than *O* lest it encourage *O* to overinvest in search.

46 Whilst this would seem to make artwork an unattractive target for thieves, art theft is the world’s third largest criminal enterprise, trailing only the trade in armaments and drugs. Stolen art is also used as collateral to secure debts incurred in the drug trade. See Ekelund, Jackson and Tollison (n 38) 171, 176. Because more famous works are more difficult to fence, thieves tend to target works valued at approximately $100,000 or less: at 177. The total value of art stolen between 2008 and 2022 is estimated to be approximately £9 billion. <www.thetimes.co.uk/article/art-detectives-arthur-brand-times-luxury-j2n39n38l> accessed 31 May 2024.

47 For the seminal account, see Carol Rose, ‘Possession as the Origin of Property’ (1985) 52 U Chi L Rev 73. See also Michael JR Crawford, *An Expressive Theory of Possession* (Hart Publishing 2020) ch 3.

48 James Krier and Christopher Serkin, ‘The Possession Heuristic’ in Yun-chien Chang (ed), *Law and Economics of Possession* (CUP 2015) 149.

49 On communicative trade-offs in the language of possession, see Henry Smith, ‘The Language of Property: Form, Context, and Audience’ (2003) 55 Stan L Rev 1105, 1125.

50 Thomas Merrill, ‘Possession as a Natural Right’ (2015) 9 NYU Journal of Law and Liberty 345, 358.

51 Benito Arruñada, *Institutional Foundations of Impersonal Exchange: Theory and Policy of Contractual Registries* (University of Chicago Press 2012) 25. See also Henry Hansmann and Reinier Kraakman, ‘Property, Contract, and Verification: The Numerus Clausus Problem and the Divisibility of Rights the Evolution of Property Rights’ (2002) 31 JLS S373, S384–5.

52 Richard Epstein, ‘Notice and Freedom of Contract in the Law of Servitudes’ (1981) 55 S Cal L Rev 1353, 1355. Seel also Arruñada (n 51) 25.

53 See Epstein, ‘Inducement’ (n 8) 2.

54 (1601) 3 Co Rep 80 b; 76 ER 809. See further Douglas Baird and Thomas Jackson, ‘Information, Uncertainty, and the Transfer of Property’ (1984) 13 JLS 299, 302.

55 RM Goode, ‘The Modernisation of Personal Property Security Law’ (1984) 100 LQR 234, 238. See also Douglas Baird and Thomas Jackson, ‘Possession and Ownership: An Examination of the Scope of Article 9’ (1982) 35 Stan L Rev 175, 180.

56 Baird and Jackson, ‘Information’ (n 54) 303; Epstein, ‘Notice and Freedom’ (n 52) 1355. Tellingly, English law does not recognise a system of estates in personal property. William Swadling, ‘Property’ in AS Burrows (ed), *English Private Law* (3rd edn, OUP 2013) 173, 267 [4.130].

57 Bell and Parchomovsky (n 10) 255–6.

58 As they explain, ‘just as a registry conveys (and potentially certifies) information, it necessarily denies and discredits other information that is inconsistent with the information contained in the registry’. ibid 256.

59 Unlike the ‘person of principle’, *homo economicus* is indifferent between purchasing an unimpeachable title and a defective title so long as the price for the latter has been appropriately discounted.

60 See also Shavell, ‘Foundations’ (n 33) 48. Shavell also notes that a register increases *T*’s chances of conviction because he cannot plausibly claim ownership of the goods: at 47.

61 See also Henry Smith, ‘The Elements of Possession’ in Yun-chien Chang (ed), *Law and Economics of Possession* (CUP 2015) 65, 85; Shavell, ‘Foundations’ (n 33) 49; Baird and Jackson, ‘Information’ (n 54) 306.

62 Shavell, ‘Foundations’ (n 33) 49–50. Thus, even if technological innovation made checking the ‘milk owners’ register’ extraordinarily cheap for *P*, the value of the additional gains made possible by the register would not justify the costs of establishing and maintaining it.

63 Bell and Parchomovsky (n 10) 280.

64 ibid 274.

65 ibid 267–8.

66 ibid 268–9.

67 On the trade-offs between different titling systems, see Benito Arruñada and Nuno Garoupa, ‘The Choice of Tilting System in Land’ (2005) 48 JLE 709.

68 The difference between systems of recordation and registration is that between a system in which the state is a mere recorder of rights and one in which it is the ‘arbiter of titles’. See Bell and Parchomovsky (n 10) 279. The Australian ‘Torrens’ system of land registration is the archetypal example of the latter type. In Barwick CJ’s oft-quoted words, ‘The Torrens system … is not a system of registration of title but a system of title by registration’. *Breskvar v Wall* (1971) 126 CLR 376, 385. It is thus sometimes remarked that registration operates as a new Crown grant of the registered interest. See Brendan Edgeworth, *Butt’s Land Law* (7th edn, Lawbook Co 2017) 831.

69 This is the Achilles’ heel of the Torrens system. Paradigmatically, one co-owner forges the other’s signature on a memorandum of mortgage and absconds with the advance made by the mortgagee. Because the mortgagee’s carelessness does not amount to statutory fraud, the mortgage remains ‘indefeasible’, allowing the mortgagee to exercise its power of sale and recover the proceeds of an advance that the victim never received. The leading case of *Frazer v Walker* [1967] 1 AC 569 is one of many examples of this phenomenon. The moral hazard created by the ‘indefeasibility’ of the mortgagee’s registered interest was sufficiently serious to justify statutory amendment to the Torrens legislation. The amendment conditioned the indefeasibility of a registered mortgage on proof that the mortgagee took reasonable steps to verify the mortgagor’s identity. See eg Real Property Act 1900 (NSW), s 56C.

70 For instance, users of Australia’s Personal Property Securities Register are charged fees on a ‘cost recovery’ basis. Personal Property Securities Act 2009 (Cth), s 190. See *Review of the Personal Property Securities Act 2009, Final Report*, 237 <www.ag.gov.au/legal-system/publications/review-personal-property-securities-act-2009-final-report> accessed 28 May 2024.

71 Although the presence of ‘tie-ins’ means that the inability of the private market to supply such services is often overstated. See, famously, RH Coase, ‘The Lighthouse in Economics’ (1974) 17 JLE 357.

72 See Julian Radcliffe, ‘The Art Loss Register’ (2004) 29 Art Libraries Journal 37.

73 <www.artloss.com/about-us/> accessed 31 May 2024.

74 <www.thewatchregister.com/about-the-watch-register/> accessed 31 May 2024.

75 <www.artloss.com/register/> accessed 31 May 2024.

76 <www.artloss.com/search/> accessed 31 May 2024. As part of its Cultural Heritage at Risk service, Art Loss offers its services free of charge to nation states. See <www.artloss.com/chard/> accessed 31 May 2024.

77 <www.artrecovery.com/services> accessed 31 May 2024.

78 Due to strong network effects, the total number of such registers will be small. A service will be subject to a network effect if its users enjoy positive returns from increased use. A good example is a social media platform. See Michael Katz and Carl Shapiro, ‘Network Externalities, Competition and Compatibility’ (1985) 75 American Economic Review 424, 424–5.

79 Steven Grover, ‘The Need for Civil-Law Nations to Adopt Discovery Rules in Art Replevin Actions: A Comparative Study’ (1991) 70 Tex L Rev 1431, 1437; Steven Bibas, ‘The Case against Statutes of Limitations for Stolen Art’ (1993) 103 Yale LJ 2437, 2461 fn 141; Dea Sula, ‘The Need for a United States National Antiquities Database: A Valuable Suggestion from the Nicosia Convention’ (2023) 22 Santa Clara Journal of International Law 29, 44.

80 The accuracy of such beliefs is a different question. An alternative, and more innocuous, explanation for the reticence to register is that owners do not wish to broadcast their ownership of valuable assets to potential thieves.

81 Some commentators recognise that registration depresses demand for stolen art but fail to appreciate that *O*’s interests will differ depending upon whether her art is merely at risk of being stolen or has actually been stolen. See eg Grover (n 79) 1436.

82 569 NE2d 426 (NY 1991); 77 NY2d 311.

83 ibid 318–19.

84 ibid 320.

85 For a similar observation, see Bibas (n 79) 2459–60. Unsurprisingly, Bibas criticises the Court’s decision in *Lubell* to adopt the ‘ex post’ perspective: at 2459.

86 For an extended treatment, see Anja Shortland, *Kidnap: Inside the Ransom Business* (OUP 2019).

87 See also Bibas (n 79) 2459.

88 ibid 2460.

89 ibid 2466.

90 And even systems of registered title, as opposed to title *by* registration, cannot assure *P* that the registered instrument is effective. A system of title by registration in which the state guarantees the registered title must be implemented by the state.

91 Yun-chien Chang, ‘The Good-Faith Purchase Doctrine in 247 Jurisdictions’ (2020) 9 European Property Law Journal 133, 147. See also Dari-Mattiacci and Guerriero (n 16) 545–6.

92 Saul Levmore, ‘Variety and Uniformity in the Treatment of the Good-Faith Purchaser’ (1987) 16 JLS 43. See also Boris Kozolchyk, ‘Transfers of Personal Property by a Nonowner: Its Future in Light of Its Past’ (1986) 61 Tul L Rev 453. This does not undermine Smith’s observation that all systems establish *nemo dat* as the default position because, as Smith notes, the existence of a common starting point does not determine how extensive or numerous any exceptions to it should be. Henry Smith, ‘On the Economy of Concepts in Property’ (2012) 160 U Pa L Rev 2097, 2122–3.

93 Weinberg (n 12) 586.

94 See eg art 1153 of the Italian Civil Code, which provides that *P* is always preferred to *O*. According to Chang, Italian, Hungarian and Guatemalan law are the most generous to *P*. Chang, ‘Good-Faith Purchase’ (n 91) 145.

95 Rogers (n 37) 481–2.

96 Epstein, ‘Inducement’ (n 8) 12–13.

97 In McFarlane’s neat summation, the effect of any given rule is simply to ‘trade retrospective security … for prospective insecurity’, or *vice versa*. McFarlane (n 9) 189. One exception might be the preferential treatment that the law appears to afford to repeat commercial players such as mercantile agents and those who buy from them. However, unlike sales in market overt, these exceptions do not extend to the sale of stolen goods because they typically require that the agent be in possession with the owner’s consent. See eg Factors Act 1889, s 2(1).

98 This belies Blackstone’s claim that the market overt exception to *nemo dat* is essential for the maintenance of commerce. William Blackstone, *Commentaries on the Laws of England. Book II, Of the Rights of Things* (OUP 2016) 304. So long as there is theft, its costs must either be borne by *P*, under a *nemo dat* rule, or *O*, under a rule that favours good faith purchasers. Because *P* becomes *O* the moment title passes, he should favour the rule with the lowest *expected* costs. For essentially the same observation, see Levmore, ‘Variety’ (n 92) 55. Levmore suggests that a better justification for the market overt rule is that it aids law enforcement by channelling sales into public and stationary markets: at 55. This conjecture is consistent with the requirement that sales in market overt be conducted in public during daylight hours and that the goods be of a type typically sold in that market. See JG Pease, ‘Market Overt in the City of London’ (1915) 31 LQR 270, 276–7, 283; Daniel Murray, ‘Sale in Market Overt’ (1960) 9 ICLQ 24, 26; Smith, ‘Valediction’ (n 34) 228, 235–6. Whilst the market overt exception protected *P* from a claim by *O*, the vendor was still liable to *O* in conversion. See Smith, ‘Valediction’ (n 34) 234.

99 Epstein, ‘Inducement’ (n 8) 13. Medina thus argues that the optimal rule is one that induces the highest expected value of the ‘ownership right’, which consists of *O*’s reservation value and *P*’s willingness to pay. See Medina (n 12) 348.

100 On these trade-offs, see Medina (n 12) 368.

101 Levmore, ‘Variety’ (n 92) 45. Or, as Medina concludes, that optimising the expected value of ownership is impossibly difficult. Medina (n 12) 369.

102 Because insurers must calculate the premium according to the average risk of members of the insurance pool, this externality problem cannot be solved by simply increasing the premium. See MV Pauly, ‘The Economics of Moral Hazard’ (1968) 58 American Economic Review 531, 534. The inability to charge premiums on the margin ultimately threatens the problem of ‘adverse selection’.

103 This is just a restatement of the famous ‘Coase Theorem’. See RH Coase, ‘The Problem of Social Cost’ (1960) 3 JLE 1.

104 See Richard Posner, ‘The Value of Wealth: A Comment on Dworkin and Kronman’ (1980) 9 JLS 243, 243.

105 See Richard Epstein, ‘The Clear View of the Cathedral: The Dominance of Property Rules’ (1996) 106 Yale LJ 2091, 2094. Rose distinguishes between ‘Type I’ and ‘Type II’ bargaining costs. The former are the familiar costs of assembly, whereas the latter are those caused by strategic bargaining in thin markets. Carol Rose, ‘The Shadow of The Cathedral’ (1997) 106 Yale LJ 2175, 2183–4. The problem of strategic bargaining is attributable to the absence of a ‘natural’ rule for dividing the surplus. In contrast to the Coase Theorem, Cooter calls this the ‘Hobbes Theorem’. Robert Cooter, ‘The Cost of Coase’ (1982) 11 JLS 1, 18.

106 Polinsky observes that, when viewed through the lens of efficiency, the danger caused by holdouts is one of ‘unsuccessful extortion’. A Mitchell Polinsky, ‘Resolving Nuisance Disputes: The Simple Economics of Injunctive and Damage Remedies’ (1980) 32 Stan L Rev 1075, 1078.

107 Dari-Mattiacci and Guerriero argue that, at least in ‘developed markets’, *P* will value the good more highly than *O*. Dari-Mattiacci and Guerriero (n 16) 549. It is not obvious why this is true.

108 This is because the only explanation for bailing goods to a mercantile agent for sale is that the bailor values the goods at less than their market value. If the law awarded the goods to *O*, then, absent transactions costs, *O* would simply sell them back to *P*. Exceptions to *nemo dat* for sales by mercantile agents, such as that contained in s 2(1) of Factors Act 1889, obviate the need for this additional transaction. See Richard Posner and William Landes, ‘The Economics of Legal Disputes Over the Ownership of Works of Art and Other Collectibles’ (1996) Coase-Sandor Institute for Law & Economics Working Paper No 40, 45–6, <https://chicagounbound.uchicago.edu/law_and_economics/513/> accessed 26 September 2024.

109 If bargains were costless and information free, it would be tautologically true that rights to all resources would invariably reside with their highest-valuing user. Once again, this is just a restatement of the Coase Theorem.

110 One is subject to the endowment effect if the price that one would accept as vendor exceeds the price one would be prepared to pay as purchaser. It is thus an example of intransitive preferences. See generally Francesco Parisi, *The Language of Law and Economics* (CUP 2013) 103–4.

111 Daniel Kahneman, Jack Knetsch and Richard Thaler, ‘Experimental Tests of the Endowment Effect and the Coase Theorem’ (1990) 98 Journal of Political Economy 1325, 1342.

112 Calabresi and Melamed (n 45) 1105–6.

113 ibid 1106–7.

114 Ian Ayers, *Optional Law: The Structure of Legal Entitlements* (University of Chicago Press 2005), 5.

115 On the potential for a liability rule to under-compensate a right holder, see Epstein, ‘The Clear View’ (n 104) 2093.

116 Discussed in Levmore, ‘Variety’ (n 92) 52. For a modern example, see art 1714 of the Civil Code of Québec.

117 The court would presumably apply the procedures that govern the sale of collateral by the holder of an equitable lien.

118 Gideon Parchomovsky, Peter Siegelman and Steve Thel, ‘Of Equal Wrongs and Half Rights’ (2007) 82 NYU L Rev 738, 744.

119 Parchomovsky, Siegelman and Thel remark that, ‘In cases where nobody has a superior claim to a positive or negative windfall, it is almost tautological that the fair outcome is to divide the windfall evenly between the contesting parties’. ibid 761.

120 Levmore, ‘Variety’ (n 92) 63–5.

121 ibid 64.

122 If the market for the disputed item were thick, there would be no temptation for the favoured party to hold out and thus no need for judicial valuation in the first place.

123 Chang also uses divided ownership to ensure allocative efficiency. Chang’s solution relies on developments in mechanism design which demonstrate that, if no individual is allocated more than 79% of the disputed asset, the parties will bargain to the allocatively efficient solution. Largely for reasons of fairness, Chang proposes that each party be allocated a 50% share. See Chang, *Property Law* (n 28) 283–4. Chang restricts ‘internal auctions’ to cases in which *O* and *P* have invested optimally in precautions and verification, respectively: at 285. By contrast, the argument made here is that a departure from the winner-takes-all procedure will invariably apply in the absence of a register. The reason for not adopting Chang’s coda is that, as with Schwartz and Scott’s negligence solution, the cost of determining whether the parties took efficient precautions is prohibitive.

124 See William Vickrey, ‘Counterspeculation, Auctions, and Competitive Sealed Tenders’ (1961) 16 Journal of Finance 8.

125 A dominant strategy is one that a player adopts *irrespective* of the strategy adopted by the other player(s).

126 Vickrey (n 124) 20–1.

127 See Lee Anne Fennell, ‘Revealing Options’ (2004) 118 Harv L Rev 1399, 1429–31; Saul Levmore, ‘Self-Assessed Valuation Systems for Tort and Other Law’ (1982) 68 Va L Rev 771, 838–9; Ian Ayres and Eric Talley, ‘Solomonic Bargaining: Dividing a Legal Entitlement to Facilitate Coasean Trade’ (1994) 104 Yale LJ 1027, 1072.

128 For discussion of this point, see Fennell (n 127) 1411, 1418. Ayres and Talley refer to this as the salutary effect of an ‘identity crisis’: Ayres and Talley (n 127) 1030.

129 Imagine that *O* has a reserve price of £1000 and *P* has a reserve price of £2000. If *O* is selected a valuer and nominates a price of £1000, then *P* will exercise her call option to purchase *O*’s share and will capture the entire £1000 surplus. If, on the other hand, *P* is selected as valuer and nominates a value of £2000, *O* will exercise his put option, forcing *P* to purchase *O*’s share at *P*’s price, in which case *O* will capture the entire £1000 surplus. As Levmore notes, ‘it is disadvantageous to reveal one’s preferences when another party has the subsequent right to choose’. Levmore, ‘Self-Assessed Valuation’ (n 127) 842. Unfortunately, the Vickrey auction cannot solve this problem because ‘truth’ ceases to be a dominant strategy when the winning party pays the average of the high and low bids.

130 Parchomovsky, Siegelman and Thel (n 118) 788.

131 Vickrey (n 124) 14. A sealed bid auction in which the highest bidder pays his own bid is conceptually equivalent to a descending price, or ‘Dutch’, auction.

132 Strictly speaking, one should distinguish different types of ascending auction. The Vickrey auction is identical only to the ‘Japanese’ auction, in which the price continually rises and no ‘jump bids’ are permitted. See Paul Klemperer, *Auctions: Theory and Practice* (Princeton UP 2018) 11–12.

